# Carboplatin Plus Vincristine as an Alternative Chemotherapeutic Scheme in Patients With Glioblastoma

**DOI:** 10.7759/cureus.24467

**Published:** 2022-04-25

**Authors:** Marcos V Sangrador-Deitos, Eliezer Villanueva-Castro, Ricardo Marian-Magaña, Luis A Rodríguez-Hernández, Gerardo Y Guinto-Nishimura, Juan L Gómez-Amador, Teresa Corona-Vázquez, Talia Wegman-Ostorozky, Sonia Mejia

**Affiliations:** 1 Department of Neurosurgery, Instituto Nacional de Neurología y Neurocirugía Manuel Velasco Suárez, Mexico City, MEX; 2 Department of Neurology, Instituto Nacional de Neurología y Neurocirugía Manuel Velasco Suárez, Mexico City, MEX; 3 Department of Genetics, Instituto Nacional de Cancerología, Mexico City, MEX

**Keywords:** vincristine, radiotherapy, glioblastoma, chemotherapy, carboplatin

## Abstract

Background and objective

Alternative chemotherapy regimens, including cisplatin, carmustine, or other agents, have been shown to be effective; however, the use of carboplatin plus vincristine (C/V) has not been studied before. In this study, we aimed to determine the survival rates in patients treated with C/V, by comparing our findings with treatments based on temozolomide (TMZ), and to explore a possible relationship with the methylation status of the methylguanine methyltransferase (MGMT) promoter in patients with glioblastoma (GB).

Methods

A retrospective cohort study was conducted involving 45 surgically treated patients diagnosed with GB. Fresh tissue samples were examined by the DNA bisulfite conversion method to determine methylation status. After surgery, different chemotherapy regimens were employed as adjuvants. Follow-up of participants was performed as outpatients at three-month intervals to determine overall survival (OS), by comparing the use of TMZ versus C/V.

Results

MGMT promoter methylation status could only be determined in 35 samples; 20 patients received adjuvant chemotherapy, of which 14 were treated with C/V and six with TMZ-based schemes. The median OS (mOS) was eight months (range: 1-24 months). OS was 57.25% at six months, 48.7% at 12 months, and 28.5% at 24 months. In the TMZ group, an OS of 83% was observed at 24 months. In the C/V group, OS was 71.4% at six months, 57.1% at 12 months, and 35.7% at 24 months. Patients who did not receive adjuvant chemotherapy treatment had the lowest survival rates with an OS of 39.9% at six months, 26.6% at 12 months, and 19.9% ​​at 24 months.

Conclusions

Based on our findings, C/V offers an accessible and effective alternative treatment when the TMZ-based scheme is not accessible, providing higher rates of OS compared to patients without chemotherapy management. The methylation status of the MGMT promoter is a significant prognostic factor, resulting in higher survival rates among patients when it is methylated.

## Introduction

According to the World Health Organization (WHO), cancer is the first or the second leading cause of mortality in developed countries, with brain cancer being one of the most morbid conditions among them [1]. Based on projections made by the GLOBOCAN database, it is estimated that the total number of cases shall rise by more than 50,000 by 2030 [2]. Regarding central nervous system (CNS) tumors, glioblastoma (GB) stands out as the most common primary neoplasm [1]. These lesions initially present with non-specific symptoms, such as headache in 53-57% of individuals and seizures in 23-56% [3]; however, the clinical presentation can vary depending on the location of the tumor.

Nowadays, GB treatment consists of surgery, accompanied by radio and chemotherapy [3]. Previous studies have demonstrated that the extent of resection (EOR) during surgery is one of the most significant prognostic factors, as it has a direct effect on patients' overall survival (OS) when total and partial macroscopic resections have been compared [4-7]. Adjuvant therapy consists of 60-Gy fractionated radiotherapy (29-30 fractions of 1.8-2.0 Gy each) [8-10] plus temozolomide (TMZ)-based chemotherapy. Although guidelines propose TMZ as the first-line treatment, economic constraints and lack of availability can be major obstacles to its administration in routine practice, necessitating the use of alternative agents [8] such as carboplatin plus vincristine (C/V). Carboplatin is an alkylating agent that acts via three different mechanisms: incorporation of alkyl groups (methyl) to DNA, formation of crossed-links in DNA, and induction of nucleotide miss-pairing [11]. Vincristine is an alkaloid agent, which bonds to the mitotic spindle, specifically to tubulin's beta chain, and blocks metaphase, causing cellular death [12].

The methylguanine methyltransferase (MGMT) gene is located in chromosome 10q26, and it codes for the 207 aminoacids O6-MGMT protein. This enzyme acts as a DNA-repairing mechanism, irreversibly transferring a methyl group from the O6 position of the DNA's guanine nucleotide to a cysteine terminal of the MGMT protein. This mechanism of action prevents cell death induced by alkylating agents, such as TMZ and carboplatin. MGMT's promoter methylation neutralizes this mechanism, causing diminished DNA-repairing activity [13]. Clinically, this implies that those cells with high MGMT expression are usually chemoresistant, while those with low expression are chemosensitive. However, this is not a rule [14].

Previous investigations conducted at our institution have revealed that the mean age at diagnosis is 45.7 years, with 6.5% of the patients categorized as WHO grade I glioma, 12.3% as grade II, 23.2% as grade III, and 58% as grade IV. Only 40% received chemotherapy, and only 10% of those received TMZ, while the rest received alternative schemes based on C/V [15].

The main objective of this article is to describe the survival rates in patients with GB who were treated with an alternative chemotherapy regimen based on C/V, in order to demonstrate the superiority of this treatment compared to patients who do not receive any chemotherapy regimen and to compare these results with regimens based on TMZ. With regard to secondary objectives, the survival rates of patients who received adjuvant radiotherapy were compared with those who did not receive such treatment, and the MGMT promoter methylation status was analyzed.

## Materials and methods

We conducted a retrospective study in which 45 patients with GB were included. These patients were recruited over a one-year period. The clinical records and complementary studies of these patients were analyzed in detail. The main inclusion criteria were as follows: patients with a histological diagnosis of GB certified by two expert neuropathologists, those aged over 18 years, and those without any prior radiotherapy or chemotherapy. All patients signed informed consent for all the procedures described in this paper.

To determine the methylation status of the MGMT promoter, the DNA bisulfite conversion method (Kit AB117126 - Bisulfite Conversion Kit - Whole Cell) and methylation-sensitive polymerase chain reaction (PCR) were performed on all samples. Bisulfite conversion is a process in which DNA is denatured and treated with sodium sulfate, leading to the deamination of non-methylated cytosines to uracils, while methylated cytosines remain unchanged. The DNA is amplified by PCR where the uracils are converted to thymines. This converted DNA can be analyzed to differentiate between methylated and non-methylated sequences and provide a methylation profile of the sample. DNA from peripheral blood lymphocytes was used as a negative control. As a positive control, in vitro methylation of lymphocytes was performed with CpG methyltransferase (M. Sssl), which is an enzyme that completely methylates all cytosine residues in double-stranded, non-methylated, and hemimethylated DNA of 5'-C-phosphate-G-3' (CpG islands). These blood samples were obtained from a healthy control population.

All patients were surgically treated by using different approaches depending on the location of the tumor. After surgery, patients were followed up on an outpatient basis for 24 months at three-month intervals, through clinical examination and contrast-enhanced MRI. Demographic and clinical variables of the population were documented. Continuous variables were summarized as means or medians and categorical variables were reported as percentages.

OS was evaluated for all patients, calculated from the day of surgery, leading to histological diagnosis. Data were analyzed using SPSS Statistics V 21.0 (IBM, Armonk, NY). Kaplan-Meier curves for survival analysis were obtained using log-rank tests. A p-value ≤0.05 was considered statistically significant.

## Results

Patient characteristics and treatments applied

In Table 1, the characteristics of the patients are listed and broken down according to the different study groups assigned. One patient presented with functional deterioration with worsening of the Karnofsky Performance Scale (KPS) scores secondary to refractory cerebral edema. Twenty patients received adjuvant chemotherapy, of which 14 were treated with C/V, and six with TMZ-based schemes. Data on surgical, radiotherapy, and chemotherapy treatment as well as MGMT methylation status are summarized in Tables 2-4.

**Table 1:** Demographic and clinical data

Characteristics	Values (n=35)
Sex, n (%)	
Male	19 (54)
Female	16 (46)
Mean age (years)	53.31
Tumor location, n (%)	
Temporal	15 (43)
Frontal	11 (31.5)
Parietal	6 (17.2)
Occipital	1 (2.9)
Other	2 (5.4)
Initial symptoms, n (%)	
Headache	11 (41.4)
Cognitive impairment	8 (23)
Seizure	7 (20)
Motor symptoms	7 (20)
Sensitive symptoms	1 (3)

**Table 2:** Surgical results C/V: carboplatin plus vincristine; EOR: extent of resection (as calculated using MRI volumetric measurements in Brainlab's software Elements^TM^ SmartBrush); Postop: postoperative; Preop: preoperative; KPS: Karnofsky Performance Scale; TMZ: temozolomide

Characteristics	Total patients (n=35), n (%)	C/V (n=14), n (%)	TMZ (n=6), n (%)	None (n=15), n (%)
EOR				
Total (≥95%)	21 (60)	9 (64.2)	4 (66.6)	8 (53.2)
Subtotal (<95%)	14 (40)	5 (35.8)	2 (33.4)	7 (46.8)
Preop KPS score				
≥70	31 (88.6)	12 (85.6)	6 (100)	13 (86.5)
<70	4 (11.4)	2 (14.4)	0 (0)	2 (13.5)
Postop KPS score				
≥70	30 (85.7)	12 (85.6)	6 (100)	12 (80)
<70	5 (14.3)	2 (14.4)	0 (0)	3 (20)
Complications				
Surgical site infection	2 (5.6)	0 (0)	1 (100)	1 (100)
Hematoma	1 (3)	1 (100)	0 (0)	0 (0)

**Table 3:** Adjuvant therapy and methylation status C/V: carboplatin plus vincristine; TMZ: temozolomide

	N (%)
Radiotherapy	
Yes	19 (54.3)
No	16 (45.7)
Chemotherapy	
C/V	14 (40)
TMZ	6 (17.1)
None	15 (42.9)
Methylation status	
Methylated	4 (11.4)
Non-methylated	22 (62.9)
Hemimethylated	9 (25.7)

**Table 4:** Relationship between chemotherapy and methylation status C/V: carboplatin plus vincristine; TMZ: temozolomide

Chemotherapy	Methylation status				
	Methylated	Non-methylated			Hemimethylated
C/V	2	6			6
TMZ	0		5	1	
None	2		11	2	

Chemotherapeutic regimens

**Figure 1:**
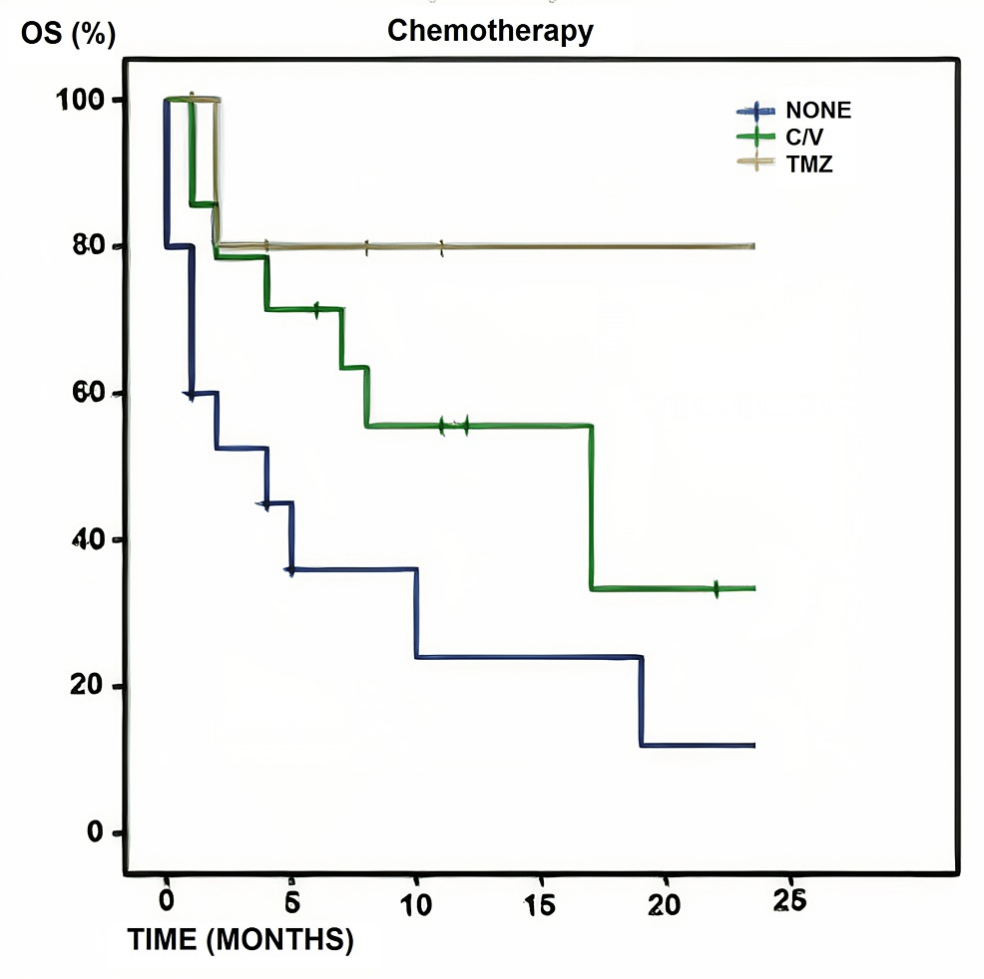
Chemotherapy and overall survival Overall survival according to chemotherapy schemes applied in 35 patients (TMZ: n=6; C/V: n=14; none: n=15). Differences between both groups were statistically significant (p=0.045) C/V: carboplatin plus vincristine; OS: overall survival; TMZ: temozolomide

Only 20 patients in the cohort received chemotherapy. Six were treated with TMZ-based first-line therapy (75 mg/m^2^ of body surface area per day, seven days a week from the first to the last day of radiotherapy followed by six cycles of 150-200 mg/m^2^ for five days every 28 days) and 14 were treated with C/V (carboplatin 450 mg and vincristine 2 mg every 28 days for at least 12 cycles). This management began at the time of the first consultation with neurological oncology, which occurred an average of two to three weeks after the surgical intervention; 15 patients could not afford any of the aforementioned chemotherapeutic agents. Survival rates largely depended on whether one or the other scheme was used. In the TMZ group, only one patient died due to non-neurological complications with an OS of 83% at 24 months. In the C/V group, OS was 71.4% at six months, 57.1% at 12 months, and 35.7% at 24 months. As expected, patients who did not receive adjuvant chemotherapy treatment had the lowest survival rates with an OS of 39.9% at six months, 26.6% at 12 months, and 19.9% ​​at 24 months. OS was higher in the TMZ-treated groups, followed by C/V (p=0.045) (Figure 1).

Overall survival and dependency on MGMT promoter methylation status

**Figure 2:**
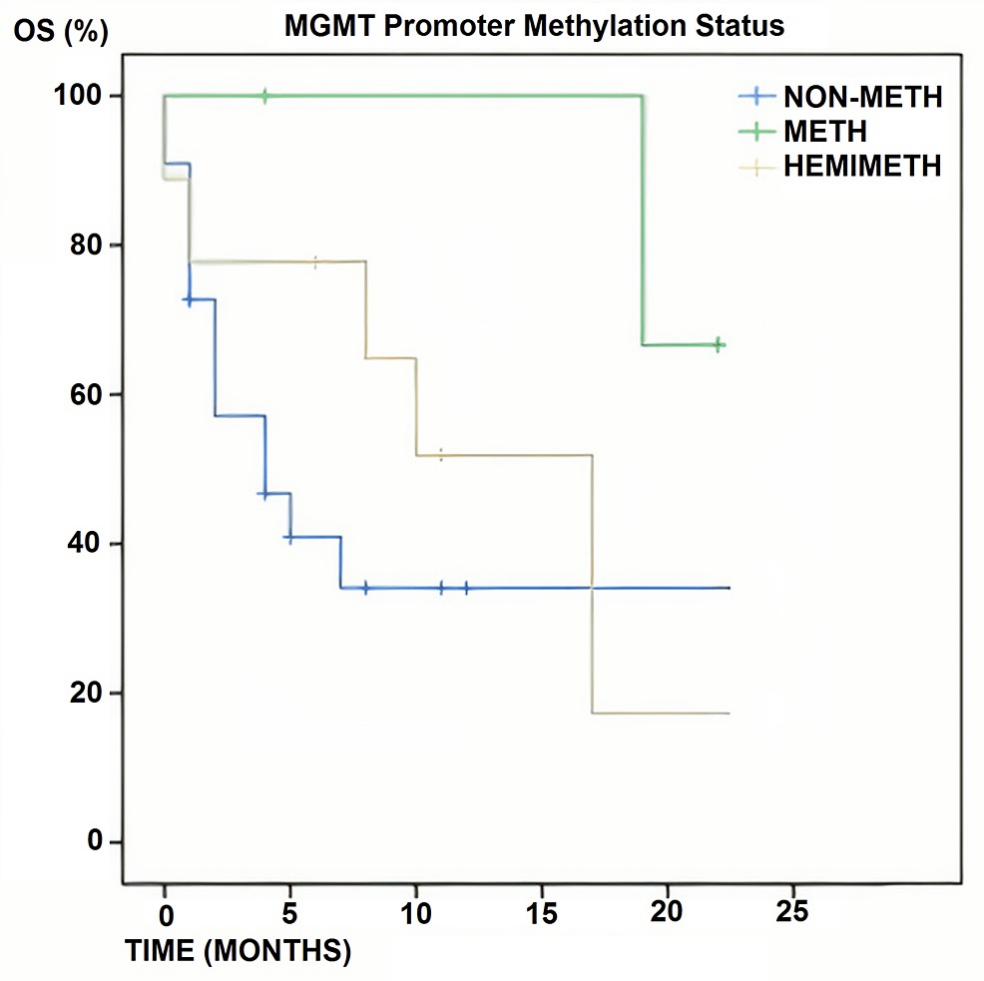
MGMT promoter methylation status Overall survival according to the MGMT promoter methylation status in 35 assessable patients (non-methylated: n=22; methylated: n=4; hemimethylated: n=9). Differences between the groups were statistically significant (p=0.012) MGMT: methylguanine methyltransferase; OS: overall survival

Overall, 45 patients were treated, obtaining 45 tumor samples. The MGMT promoter methylation status could only be determined in 35 samples, as 10 samples could not be processed properly due to technical difficulties related to sample preservation. Among the 35 patients included, the median OS (mOS) was eight months (range: 1-24 months). OS was 57.25% at six months, 48.7% at 12 months, and 28.5% at 24 months. Four of the 35 patients had a methylated MGMT promoter. Survival rates were shown to be dependent on MGMT promoter methylation status: mOS was 4.5 months for non-methylated patients, 10 months for hemimethylated, and 24 months for methylated patients (p=0.012). In the methylated group, 75% of patients were still alive at two years of follow-up, but only 36% in the non-methylated group survived (Figure 2).

Radiotherapy

**Figure 3:**
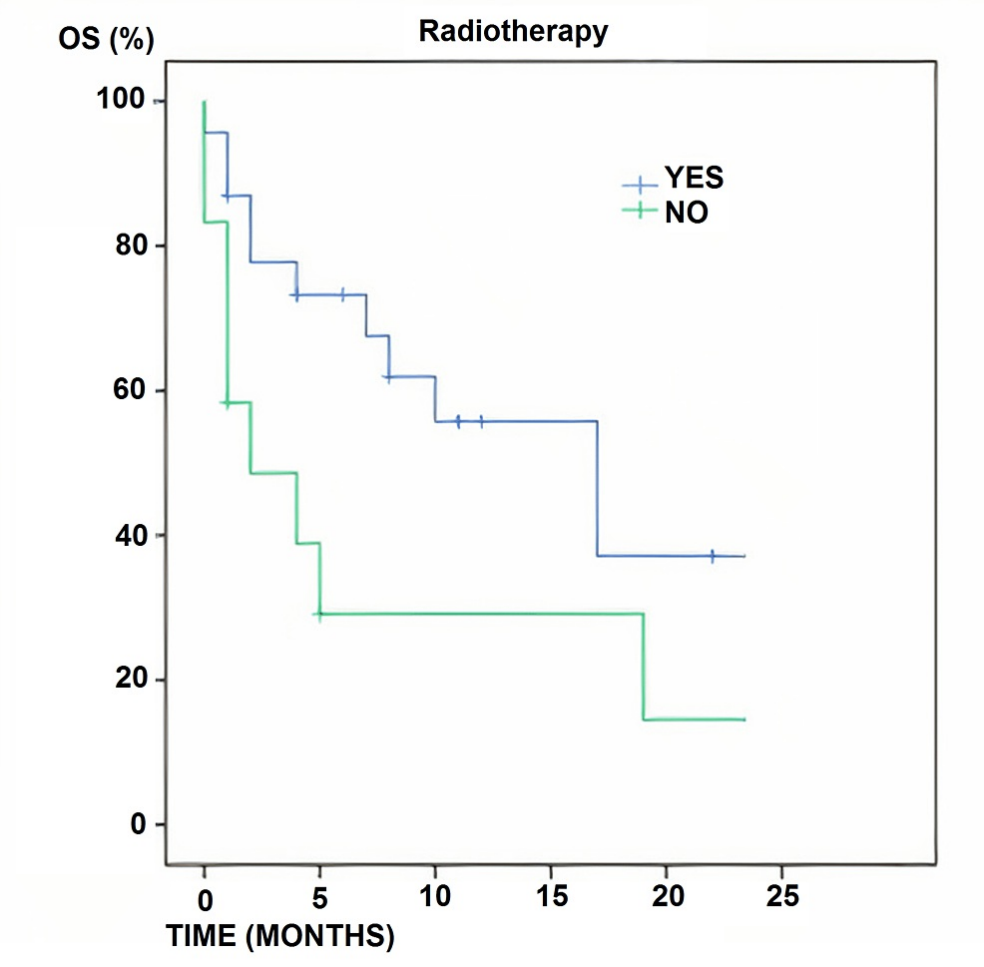
Radiotherapy and overall survival Overall survival according to whether radiotherapy was applied or not (yes: n=19; no: n=16). Differences between the groups were statistically significant (p=0.05) OS: overall survival

Radiotherapy was performed in 19 patients with a total dose of 60 Gy in single daily fractions of 2 Gy. In this group, OS was 73.6% at six months, 57.8% at 12 months, and 36.8% at 24 months, with an mOS of 17 months. When patients did not receive adjuvant radiotherapy, survival rates tended to decrease significantly with an OS of 31.2% at six months, 31.2% at 12 months, and 18.7 at 24 months, with an mOS of 2.5 months (Figure 3).

## Discussion

Even though the role of MGMT promoter methylation status as a prognostic and predictive factor in TMZ-based treatments is widely known, there are only a few studies that evaluate it as a biomarker in relation to other therapies (Table 5).

**Table 5:** Clinical implications in glioblastomas and MGMT promoter methylation status RT: radiotherapy; TMZ: temozolomide

Trial	Treatment	Patients	Progression-free survival (months)	Overall survival (months)
Esteller et al. (2000) [16]	RT, cisplatin & carmustine	49 patients with anaplastic astrocytoma or glioblastoma	21 methylated vs. 8 non-methylated	30 methylated vs. 21 non-methylated
Hegi et al. (2005)[17]	RT vs. RT & TMZ	573 patients with glioblastoma	5.9 vs. 10.3 methylated, 4.4 vs. 5.3 non-methylated	15.3 vs. 21.7 methylated, 11.8 vs. 12.7 non-methylated
Herrlinger et al. (2006)[18]	RT & TMZ followed by TMZ or lomustine	31 patients with glioblastoma	19 methylated vs. 6 non-methylated	34.3 methylated vs. 12.5 non-methylated
Weller et al. (2009)[19]	RT vs. RT & TMZ	301 patients with glioblastoma	7.5 methylated vs. 6.3 non-methylated	18.9 methylated vs. 11.1 non-methylated
Gilbert et al. (2013)[20]	RT + TMZ followed by TMZ (5/28 days) vs. RT + TMZ followed by TMZ (21/28 days)	833 patients	8.8 vs. 11.7 methylated, 7.1 vs. 8.2 non-methylated	23.5 vs. 21.9 methylated, 16.6 vs. 15.4 non-methylated

Surgical resection has been considered the cornerstone of treatment for GB, with EOR representing one of the most important prognostic factors [4,21]. A 2016 meta-analysis showed that patients undergoing gross total resection are 61% more likely to survive for one year, 19% more likely to survive for two years, and 51% more likely to be progression-free at 12 months, compared to patients treated with subtotal resection [22]. However, surgery alone cannot be considered to be curative. Since 2005, the standard-of-care treatment has changed drastically, following the publication of a clinical trial that showed that TMZ plus radiotherapy increases survival when compared with radiotherapy alone [23]. Furthermore, long-term survival also showed an increase when comparing the aforementioned groups [24]. Multiple studies have proved the efficiency of these treatment modalities in increasing survival rates [25,26], and hence this so-called “Stupp Protocol” is nowadays considered the gold standard for GB patients [27].

The data we present reaffirms the well-known fact that TMZ-based chemotherapy, MGMT promoter methylation status, and adjuvant radiation therapy result in the highest survival rates after surgical resection. Only 20 of the studied patients were able to receive some chemotherapy management, leaving 15 without any postoperative management. A large number of patients did not receive treatment, and therein lies the relevance of this study: to offer an alternative to this highly vulnerable population. The mOS of patients with a non-methylated MGMT promoter was 4.5 months, compared to 24 months in the methylated group. Glas et al. reported a median survival of 12.5 months in the non-methylated groups versus 34.5 months in the methylated groups [28]. However, in our institution, this cannot always be achieved, mainly due to economic reasons, since our patients, prior to 2020, had to cover all economic expenses by themselves, and most of them belong to low-income groups. That is why we must look for alternative treatments affordable to our population, and this could also apply to other institutions that share similar conditions. The C/V pool showed OS rates of 71.4% at six months, 57.1% at 12 months, and 35.7% at 24 months, which are similar when compared with the survival rates for TMZ as reported in 2005 by Stupp et al. [23], and clearly superior when compared to patients without chemotherapy.

Our study has a few limitations. Firstly, due to the small sample size of patients with methylated status, a clear relationship between this variable and the use of the proposed treatment could not be determined, and more studies should be carried out to accomplish the same. Secondly, this was a retrospective study and it has all the limitations inherent to its design. Finally, the main objective of this study was to determine if the proposed treatment could be used in patients for whom first-line treatments are not affordable or available and to describe the survival rates compared to patients who are treated with surgery but who do not receive adjuvant chemotherapy treatment of any kind.

## Conclusions

The C/V combination offers an affordable and effective alternative treatment against newly diagnosed GB when the first-line TMZ regimen is unaffordable, as adjuvant therapy after surgical resection, in addition to radiotherapy. The methylation status of the MGMT promoter is a significant prognostic factor, with higher survival rates associated with methylation. This study suggests that its relationship is not confined to TMZ, but applies to other alkylating agents, such as carboplatin. Further studies are needed to optimize combined C/V chemotherapy. Undoubtedly, the existing evidence demonstrating the superiority of adjuvant chemotherapy management with TMZ followed by radiotherapy makes it clear that this scheme should be the first-line treatment. However, the main objective of this study has been fulfilled, by showing that in cases where such management is not available, a common situation in developing countries like ours, there are other affordable options that can provide better outcomes.

## References

[1] Ostrom QT, Gittleman H, Liao P (2014). CBTRUS statistical report: primary brain and central nervous system tumors diagnosed in the United States in 2007-2011. Neuro Oncol.

[2] (2022). IARC: estimated number of new cases from 2020 to 2040, both sexes, age [0-85+]. https://gco.iarc.fr/tomorrow/en/dataviz/isotype.

[3] Laws ER, Parney IF, Huang W (2003). Survival following surgery and prognostic factors for recently diagnosed malignant glioma: data from the Glioma Outcomes Project. J Neurosurg.

[4] Lacroix M, Abi-Said D, Fourney DR (2001). A multivariate analysis of 416 patients with glioblastoma multiforme: prognosis, extent of resection, and survival. J Neurosurg.

[5] McGirt MJ, Chaichana KL, Gathinji M (2009). Independent association of extent of resection with survival in patients with malignant brain astrocytoma. J Neurosurg.

[6] Pichlmeier U, Bink A, Schackert G, Stummer W (2008). Resection and survival in glioblastoma multiforme: an RTOG recursive partitioning analysis of ALA study patients. Neuro Oncol.

[7] Sanai N, Berger MS (2008). Glioma extent of resection and its impact on patient outcome. Neurosurgery.

[8] Kristiansen K, Hagen S, Kollevold T (1981). Combined modality therapy of operated astrocytomas grade III and IV. Confirmation of the value of postoperative irradiation and lack of potentiation of bleomycin on survival time: a prospective multicenter trial of the Scandinavian Glioblastoma Study Group. Cancer.

[9] Keime-Guibert F, Chinot O, Taillandier L (2007). Radiotherapy for glioblastoma in the elderly. N Engl J Med.

[10] Malmström A, Grønberg BH, Marosi C (2012). Temozolomide versus standard 6-week radiotherapy versus hypofractionated radiotherapy in patients older than 60 years with glioblastoma: the Nordic randomised, phase 3 trial. Lancet Oncol.

[11] Huncharek M, Kupelnick B, Bishop D (1998). Platinum analogues in the treatment of recurrent high grade astrocytoma. Cancer Treat Rev.

[12] Gan PP, McCarroll JA, Po'uha ST, Kamath K, Jordan MA, Kavallaris M (2010). Microtubule dynamics, mitotic arrest, and apoptosis: drug-induced differential effects of betaIII-tubulin. Mol Cancer Ther.

[13] Christians A, Hartmann C, Benner A (2012). Prognostic value of three different methods of MGMT promoter methylation analysis in a prospective trial on newly diagnosed glioblastoma. PLoS One.

[14] Wick W, Weller M, van den Bent M (2014). MGMT testing--the challenges for biomarker-based glioma treatment. Nat Rev Neurol.

[15] Wegman-Ostrosky T, Reynoso-Noverón N, Mejía-Pérez SI (2016). Clinical prognostic factors in adults with astrocytoma: historic cohort. Clin Neurol Neurosurg.

[16] Esteller M, Garcia-Foncillas J, Andion E (2000). Inactivation of the DNA-repair gene MGMT and the clinical response of gliomas to alkylating agents. N Engl J Med.

[17] Hegi ME, Diserens AC, Gorlia T (2005). MGMT gene silencing and benefit from temozolomide in glioblastoma. N Engl J Med.

[18] Herrlinger U, Rieger J, Koch D (2006). Phase II trial of lomustine plus temozolomide chemotherapy in addition to radiotherapy in newly diagnosed glioblastoma: UKT-03. J Clin Oncol.

[19] Weller M, Felsberg J, Hartmann C (2009). Molecular predictors of progression-free and overall survival in patients with newly diagnosed glioblastoma: a prospective translational study of the German Glioma Network. J Clin Oncol.

[20] Gilbert MR, Wang M, Aldape KD (2013). Dose-dense temozolomide for newly diagnosed glioblastoma: a randomized phase III clinical trial. J Clin Oncol.

[21] Nitta T, Sato K (1995). Prognostic implications of the extent of surgical resection in patients with intracranial malignant gliomas. Cancer.

[22] Brown TJ, Brennan MC, Li M (2016). Association of the extent of resection with survival in glioblastoma: a systematic review and meta-analysis. JAMA Oncol.

[23] Stupp R, Mason WP, van den Bent MJ (2005). Radiotherapy plus concomitant and adjuvant temozolomide for glioblastoma. N Engl J Med.

[24] Stupp R, Hegi ME, Mason WP (2009). Effects of radiotherapy with concomitant and adjuvant temozolomide versus radiotherapy alone on survival in glioblastoma in a randomised phase III study: 5-year analysis of the EORTC-NCIC trial. Lancet Oncol.

[25] Johnson DR, O'Neill BP (2012). Glioblastoma survival in the United States before and during the temozolomide era. J Neurooncol.

[26] Poon MT, Sudlow CL, Figueroa JD, Brennan PM (2020). Longer-term (≥ 2 years) survival in patients with glioblastoma in population-based studies pre- and post-2005: a systematic review and meta-analysis. Sci Rep.

[27] DiRisio AC, Goswami H, Shi C, Yunusa I, Smith TR, Mekary RA, Broekman ML (2017). P09.53 The timing of postoperative chemoradiation treatment (Stupp Protocol) after GBM resection: a systematic review. Neuro Oncol.

[28] Glas M, Happold C, Rieger J (2009). Long-term survival of patients with glioblastoma treated with radiotherapy and lomustine plus temozolomide. J Clin Oncol.

